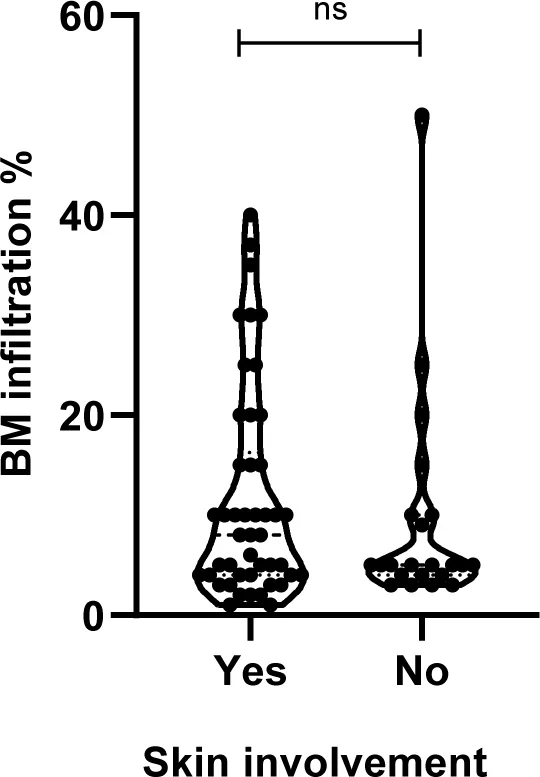# SuperRCA assay of *KIT* p.D816V mutation load reveals novel correlation to clinical phenotype

**DOI:** 10.1016/j.jacig.2026.100778

**Published:** 2026-08-18

**Authors:** Cecilia Karlström, Monika Klimkowska, Marie Engvall, Sofia Nordin, Ebba Westerberg, Sofia Papavasileiou, Joakim S. Dahlin, Johanna Ungerstedt

**Affiliations:** a Center for Hematology and Regenerative Medicine, Department of Medicine, Karolinska Institutet, Huddinge, Sweden; b ME Hematology, Karolinska University Hospital, Stockholm, Sweden; c Department of Laboratory Medicine, Karolinska Institutet, Stockholm, Sweden; d Department of Clinical Pathology and Cancer Diagnostics, Karolinska University Hospital, Stockholm, Sweden; e Rarity Bioscience AB, Uppsala, Sweden; f Department of Medicine Solna, Karolinska Institutet, Center for Molecular Medicine, and Karolinska University Hospital, Stockholm, Sweden; g Hematology Clinic, Linköping University Hospital, Linköping, Sweden; h Department of BKV, Linköping University, Linköping, Sweden

**Keywords:** KIT mutation, mast cell, systemic mastocytosis, superRCA

## Abstract

**Background:**

The *KIT* p.D816V mutation is seen in more than 90% of patients with systemic mastocytosis (SM). A high variant allele fraction (VAF) is associated with adverse outcome; however, no correlations betwen mutation burden and clinical symptoms have hitherto been established.

**Objective:**

Our aim was to quantify *KIT* p.D816V VAF in blood and bone marrow (BM) by using the ultrasensitive superRCA method and correlate the VAF with clinical symptoms.

**Method:**

A total of 107 blood and BM samples from patients with SM were analyzed.

**Results:**

In 78 of the 107 samples, a DNA input concentration of 660 ng was used to obtain a sensitivity for *KIT* p.D816V mutation of 0.0016% VAF. In paired analysis, level of *KIT* p.D816V VAF was significantly higher in BM than in blood, and it correlated with SM disease burden (ie, serum tryptase level and percentage of BM mast cell infiltration). No correlations were found between VAF and anaphylaxis, osteoporosis, or gastrointestinal symptoms. Among the patients with indolent SM (ISM), those with skin involvement had a higher blood VAF despite a similar percentage of BM mast cell infiltration.

**Conclusion:**

By using SuperRCA *KIT* p.D816V detection, we demonstrated that in the blood of patients with ISM, VAF is higher in patients with mastocytosis in the skin than in the patients without skin involvement, leading us to hypothesize that circulating *KIT* p.D816V–mutated progenitors with mast cell potential may drive the cutaneous phenotype. In addition, our findings in paired blood and BM samples support the findings of previous studies and provide further evidence supporting higher VAFs in BM than in blood as well as a correlation of VAF with disease burden.

## Introduction

Patients with systemic mastocytosis (SM) can present as having either indolent SM (ISM) or as an aggressive advanced SM (AdvSM) phenotype^1^, ^2^, ^3^, ^4^; yet, more than 90% of patients carry the activating point mutation p.D816V in their *KIT* gene. The KIT receptor is highly expressed in mast cells, rendering mutated mast cells constitutively active and proliferating and thereby causing activation symptoms from various organs, including allergic symptoms, anaphylaxis, and diarrhea or proliferation symptoms, including hepatosplenomegaly and bone marrow (BM) failure.^4^ In clinical routine, the *KIT* p.D816V mutation is measured in blood or BM by real-time quantitative PCR with a limit of detection of 0.01% mutated cells,^5^ with higher mutation burden (variant allele fraction [VAF]) linked with poor prognosis, as well as with AdvSM disease.^6^^,^^7^ Typically, VAFs are higher in BM than in blood.^5^^,^^6^^,^^8^^,^^9^ On a population level, *KIT* p.D816V VAF correlates with serum tryptase level,^6^^,^^10^ and we^11^ and others^12^ have shown that *KIT* p.D816V VAF correlates with disease burden measured as the percentage of mast cell BM infiltration.

Regarding the correlation of *KIT* p.D816V VAF with clinical symptoms, lower VAFs have been found in patients with isolated cutaneous mastocytosis than in patients with ISM,^6^ but no difference in VAFs has been found when patients with no or mild skin symptoms are compared with patients with moderate-to-severe skin symptoms, such as pruritus and flushing.^13^ A higher frequency of *KIT* p.D816V positivity has been described in patients with ISM with skin involvement than in those with ISM without skin involvement,^14^ but there are no reports of *KIT* p.D816V VAFs in patients with mastocytosis of the skin (urticaria pigmentosa) versus in patients without mastocytosis of the skin. Alvarez-Twose et al reported lower levels of serum tryptase in patients without skin involvement, also finding that the presence of *KIT* p.D816V mutations are more restricted to BM mast cells in those patients.^15^ No correlation between *KIT* p.D816V VAF and anaphylaxis has been published; however, Alvarez-Twose et al reported a lower frequency of dense compact mast cell aggregates in BM sections from patients with anaphylaxis than in BM sections from patients without anaphylaxis.^15^ However, others have found no such correlation.^13^ Overall, the relationship between *KIT* p.D816V VAF and clinical symptoms has not been investigated extensively.

The superRCA method was developed for ultrasensitive mutation detection.^16^ It has recently been used in patients with SM, proving comparable with the allele-specific oligonucleotide real-time quantitative PCR method and demonstrating higher rates of *KIT* p.D816V positivity in whole blood and/or BM in monoclonal mast cell activation syndrome and BM mastocytosis, as well as clonality in 18% of patients previously diagnosed with nonclonal mast cell activation syndrome.^17^ Mutation detection using superRCA has also been described for other targets and diagnoses (eg, *IDH1/2* mutations in patients with acute myeloid leukemia).^16^^,^^18^

We used superRCA to investigate whether this more precise measurement of *KIT* p.D816V VAF would reveal correlations to clinical symptoms. The advantage of this method is the very low limit of blank (LoB) and low limit of detection (LoD) of 0.001% VAF, as also reported by Navarro-Navarro et al^17^ if an adequate input DNA concentration higher than 660 ng is used. LoB was determined by measuring pooled DNA from 4 blood donors in a total of 30 replicates distributed over 2 days by using 1 flow cytometer, 1 operator, and 1 kit lot. The LoB was defined as the value below which 95% of the measured signals were observed. For determination of LoD, low positive samples were prepared by spiking pooled DNA from 4 blood donors with a *KIT* p.D816V–positive sample. The low positive samples were measured in a total of 30 replicates in the same manner as the LoB samples. LoD was determined as the median value of all low-positive measurements of the low-positive samples, provided at least 95% of the low-positive measurements exceeded the LoB.

Positive and wild-type controls were consistently incorporated during all assay procedures. Samples were categorized as positive if the recorded VAF percentage was above the LoD and at least 5 events were recorded. Samples with a VAF percentage above the LoB but below the LoD, as well as 5 or more mutant events, were categorized as unclassified. Samples that fulfilled neither of these criteria were categorized as testing negative.

Anaphylaxis was defined as moderate-to-severe anaphylaxis (Ring-Messmer grading ≥ II), which is in line with a recent publication by Hartmann et al.^19^ Most instances were grade III and first-time events, so no epinephrine device was used. Of the 16 patients experiencing anaphylaxis, 10 (63%) were women.

Because of nonnormal distribution of the data, nonparametric tests were used to compare the group means. The Fisher exact was used to analyze associations between 2 categoric variables. When testing group mean differences in more than 2 groups, the Kruskal-Wallis test was used. Correlations were investigated by using Spearman correlation coefficients. Missing data was handled by using pairwise deletion. This study was approved by the local ethics committee and institutional review board and was conducted in accordance with the Declaration of Helsinki.

## Results and discussion

We assessed *KIT* p.D816V VAF in 107 samples from 91 patients fulfilling SM diagnosis criteria. VAF readout was obtained from 99 of 107 samples (93%). In all, 6 samples failed analysis and 2 samples were unclassified. For 82 samples, 660 ng of DNA was obtained for DNA input and reaction, rendering the maximal sensitivity of 200,000 genomic copies, and for 33 samples, the DNA amount varied between 8 and 559 ng. Six patients (6.6%) were negative for *KIT* p.D816V mutation, which is in line with the literature.^4^ SM disease burden (measured as serum tryptase level, presence of major criterion, and percentage of BM mast cell infiltration) was higher in patients with AdvSM than in those with ISM (Table I), in line with previous reports.

**Table I:** Patient characteristics

Patient characteristics	All patients	ISM	AdvSM	*P* value
Subjects, no. (%)	91	73 (80.2)	18 (19.8)	n/a
Age (y), median (range)	59 (22-83)	53 (22-78)	73 (48-83)	∗
Male sex, no. (%)	39 (42.9)	29 (39.7)	10 (55.6)	ns
SuperRCA (VAF%), median (range)†	0.14 (0.002-41)	0.11 (0.002-26)	0.18 (0.07-41)	‡
Major criterion, no. (%)	48 (52.7)	32 (43.8)	16 (88.9)	∗
Mast cell infiltration (%), median (range)	9 (1-55)	5 (1-50)	20 (4-55)	∗
Tryptase level (μg/L), median (range)	27 (4-710)	23 (4-440)	135 (10-710)	∗
Osteoporosis/osteopenia, no. (%)	25 (27)	18 (25)	7 (39)	ns
Anaphylaxis grade ≥2, no. (%)	16 (18)	15 (21)	1 (6)	ns
Gastrointestinal symptoms, no. (%)	24 (26)	18 (25)	6 (33)	ns
Skin involvement, no. (%)	53 (58)	49 (67)	4 (22)	∗
Hb level (g/dL), median (range)	13.8 (9.2-17.2)	14.1 (9.5-17.2)	11.5 (9.2-14.1)	∗
WBC count (x 10^9^/L), median (range)	6.5 (2.7-32.1)	6.4 (2.7-15.8)	10 (2.7-32.1)	∗
Platelet count (x 10^9^/L), median (range)	285 (17-768)	289 (162-499)	261 (17-768)	ns

Because of the small sample size, the Fisher exact test was used to assess the relationship between categoric variables. The Mann-Whitney test was used to compare group means.

*Hb,* Hemoglobin; *na,* not available; *ns,* not significant; *WBC*, white blood cell.

∗ *P* < .01.

† All positive samples.

‡ *P* < .05.

**Fig 1:**
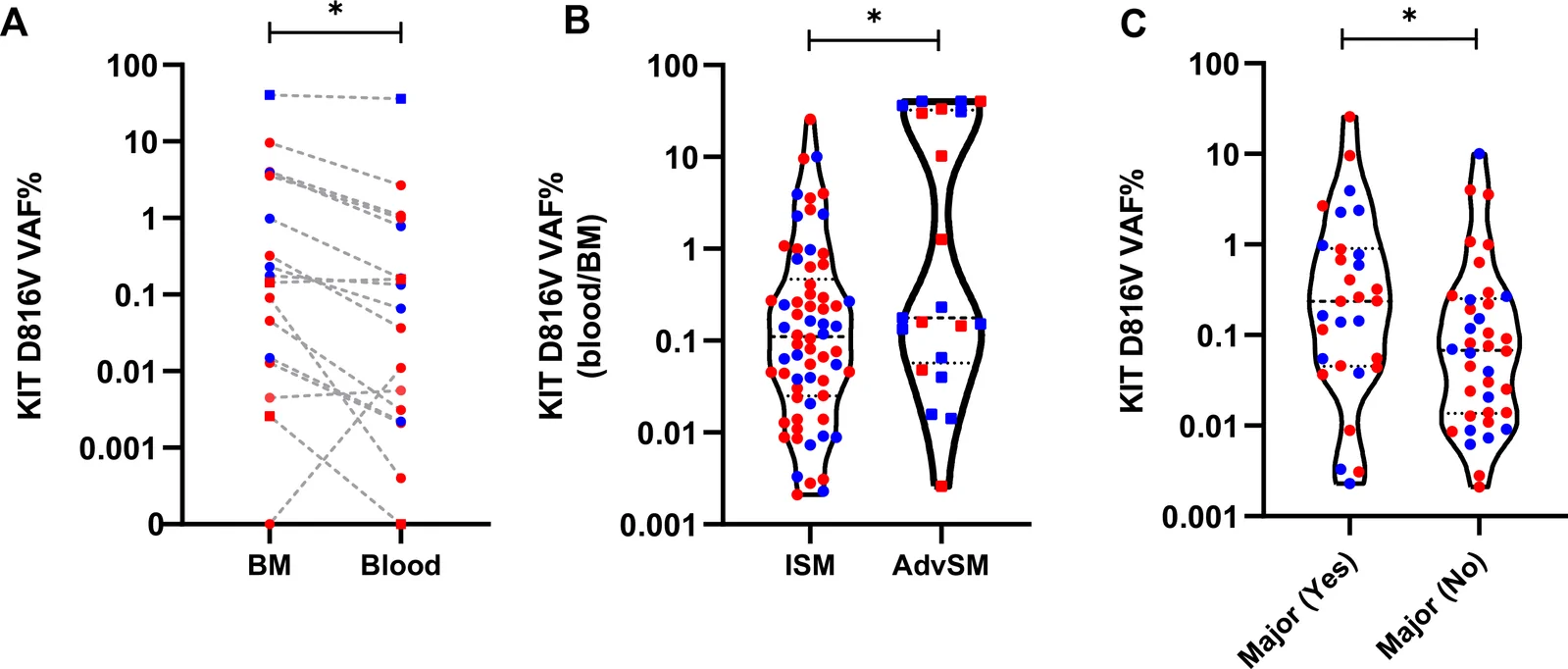
**A,***KIT* p.D816V VAF in paired samples from 17 patients with SM. **B,***KIT* p.D816V VAF in all available samples (blood and BM), patients with ISM (n = 66), and patients with AdvSM (n = 21). **C,***KIT* p.D816V VAF in ISM patients with (n = 30) or without (n = 38) the major criterion. Patients with AdvSM are marked with *squares*, and those with ISM are marked with *circles*. Colors indicate sex, with *blue* indicating men and *red* indicating women. ∗*P* < .05. *ns,* Not significant (indicates *P* > .05).

Paired BM and blood samples were available from 17 patients. In these samples, BM *KIT* p.D816V VAF was significantly higher than in blood, often reaching up to 10-fold differences (Fig 1, *A*). This difference is larger than previously reported, likely owing to more precise measurements by the superRCA method than by other methods. One patient tested negative in blood but positive in BM, and 1 patient tested negative in BM but positive in blood. Levels of *KIT* p.D816V VAF were higher in patients with AdvSM than in those with ISM (Table I and Fig 1, *B*) and higher in patients who fulfilled the major diagnostic criterion than in those who did not (Fig 1, *C*).

**Fig 2:**
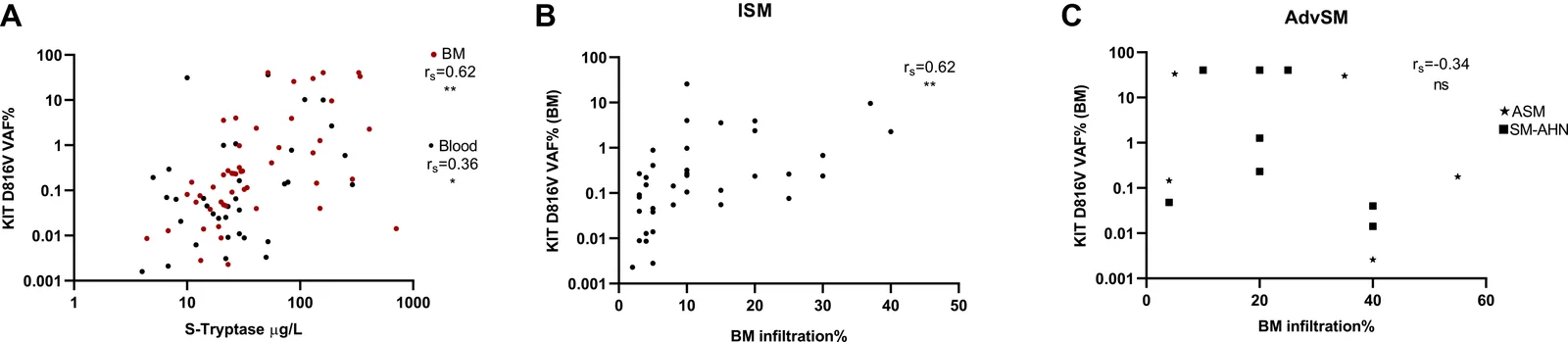
**A**, Correlation between serum tryptase level and *KIT* p.D816V VAF in BM (*red* [n = 50]) and blood (*black* [n = 35]) **B,** Correlation between *KIT* p.D816V VAF in BM (n = 39) and percentage of BM mast cell infiltration in ISM. **C**, Correlation between *KIT* p.D816V VAF in BM (n = 14) and percentage of BM mast cell infiltration in AdvSM. ∗*P* < .05; ∗∗*P* < .01. *ns*, Not significant.

Serum tryptase levels were significantly correlated with the superRCA *KIT* p.D816V–determined BM VAF (*rs* = 0.62; *P* < .001) and blood VAF (*rs* = 0.36; *P* < .05) (Fig 2, *A*).

In patients with ISM, the percentage of BM mast cell infiltration was significantly correlated with BM VAF (*rs* = 0.62; *P* < .001) (Fig 2, *B*); however, there was no correlation between percentage of BM mast cell infiltration and BM VAF for patients with AdvSM (Fig 2, *C*). Blood VAF correlated with percentage of BM mast cell infiltration in patients with ISM (*rs* = 0.56; *P* < .01) but not in patients with AdvSM (*rs* = –0.08; *P* < .89) (see Fig E1 in the Online Repository at www.jaci-global.org).

**Fig 3:**
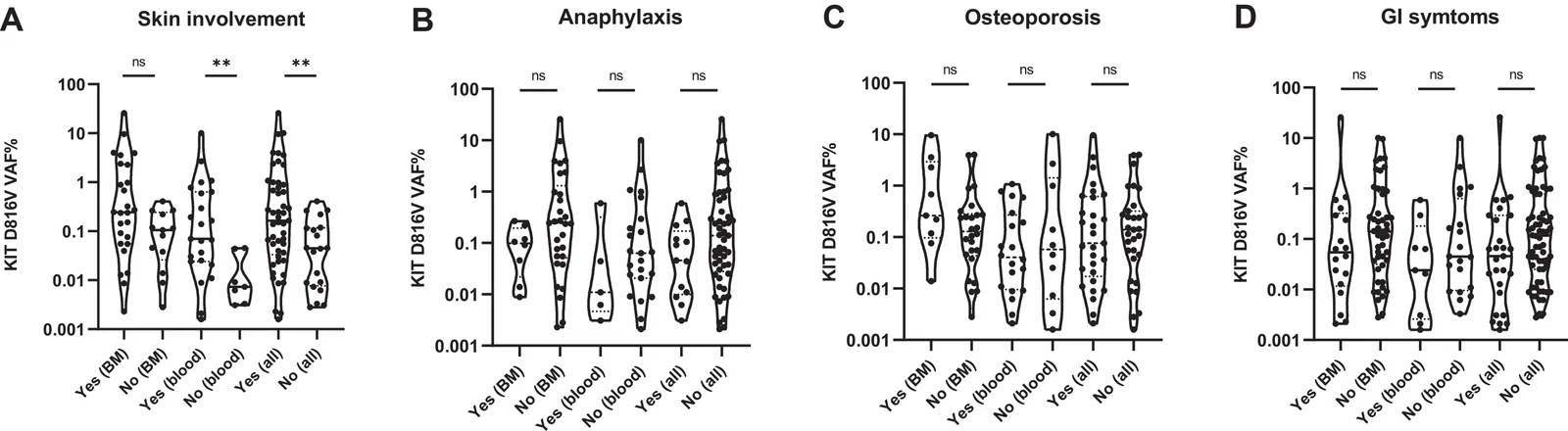
Correlation between *KIT* p.D816V VAF in blood and BM and presence of skin involvement (BM: n = 39 [yes = 26; no = 13]); blood: n = 30 [yes = 2; no = 7]) **(A)**, anaphylaxis (BM: n = 38 [yes = 8; no = 30]); blood: n = 28 [yes = 5; no = 23]) **(B)**, osteoporosis (BM: n = 35 [yes = 9; no = 26]; blood: n = 30 [yes = 20; no = 10]) **(C)**, or gastrointestinal (GI) symptoms (BM: n = 66 [yes = 18; no = 48]); blood: n = 29 [yes = 9; no = 20]) **(D)**. Yes and no indicate the presence or absence of symptoms; brackets indicate the sample type in which VAF was assessed. ∗*P* < .05; ∗∗*P* < .01. *ns,* Not significant (indicates *P* > .05).

For ISM, we assessed mutational load depending on the presence of skin mastocytosis, anaphylaxis, osteoporosis, or gastrointestinal symptoms (Fig 3). We found that VAF in blood was higher in patients with ISM with mastocytosis in the skin than in patients with ISM without mastocytosis in the skin (Fig 3, *A*), supporting previous observations of a higher frequency of *KIT* p.D816V positivity in patients with ISM with skin involvement than without skin involvement.^14^ There was no difference in percentage of BM mast cell infiltration between patients with and without mastocytosis in the skin (see Fig E2 in the Online Reposiory at www.jaci-global.org). There was a tendency toward lower VAF in patients with anaphylaxis than in those with no anaphylaxis (Fig 3, *B*), which is in line with the finding by the Orfao group of a lower frequency of dense compact mast cell aggregates in BM in patients without anaphylaxis.^15^ We found no difference in VAF depending on presence of osteoporosis or gastrointestinal symptoms (Fig 3, *C* and *D*).

We compared superRCA with clinical routine for detection of *KIT* p.D816V by using a method aligned with the reference method.^5^ A total of 33 patients were assessed with both methods in BM samples. The results for 32 of 33 patients were concordant regarding positive and negative responses. The discordant sample was positive when analyzed using superRCA (VAF 0.17 %), whereas the results were inconclusive in when analyzed using clinical routine.

For detection using samples of peripheral blood, 46 patients were tested by both methods. The superRCA method failed to yield results for 2 of the samples for reasons that we cannot fully explain. Of the 44 samples that were tested successfully, 38 had concordant test results: 6 were discordant, 2 were negative with use of superRCA and positive (near the detection limit) with use of clinical routine, and 4 were positive with use of superRCA but negative when tested according to clinical routine. Navarro-Navarro et al reported the presence of *KIT* p.D816V mutation in a proportion of patients who had previously tested negative in allele-specific oligonucleotide real-time quantitative PCR, which is in line with our findings and additionally validates the greater sensitivity of superRCA.^17^ Additional larger studies are needed to compare current *KIT* p.D816V detection methods.

We hypothesize that the higher blood VAF in patients with skin involvement indicates that circulating *KIT* p.D816V–mutated progenitors with mast cell potential may drive the cutaneous phenotype. The absence of correlation between percentage of BM mast cell infiltration and BM VAF for patients with AdvSM in this study might be due to the fact that our study population included relatively few patients with AdvSM (n = 18). The patients with AdvSM in this study comprised a very heterogenous group, with their conditions ranging from essential thrombocythemia with a good prognosis and normal life expectancy to aggressive SM with short median survival, which might be another possible explanation for the absence of correlation.

In conclusion, confirming the results reported by Navarro-Navarro et al^17^ in our independent cohort, ultrasensitive detection of *KIT* p.D816V mutation demonstrated higher VAFs in BM than in blood, and in rare cases, detection in BM but not in blood. Therefore, in selected cases, BM investigation may be performed despite a negative result of testing for *KIT* p.D816V mutation in blood. In 1 of 33 patients, superRCA yielded a positive result, whereas the result provided using our clinical routine method was inconclusive. By using superRCA, we were able to demonstrate correlations between VAF and disease burden measured as serum tryptase level and percentage of BM mast cell infiltration, which is in line with the findings of previous publications. Blood *KIT* p.D816V VAF was higher in those patients with ISM with mastocytosis in the skin than in those without skin involvement. We hypothesize that *KIT* p.D816V–mutated progenitors in blood may home to skin and cause the cutaneous phenotype.

**Key messages**

• SuperRCA *KIT* p.D816V detection provides further evidence supporting higher VAFs in BM than in blood.
• In patients with ISM, those with skin involvement had higher blood VAFs despite similar marrow burden.

## Disclosure statement

Supported by grants from Region Stockholm (to C.K.), the Center for Innovative Medicine (to C.K.), the Swedish Research Council (to J.S.D.), and Cancerfonden (to J.S.D.).

Disclosure of potential conflict of interest: M. Engvall and S. Nordin are employees of Rarity Biosciences. The rest of the authors declare that they have no relevant conflicts of interest.

## Figures and Tables

**Supplementary Figure S1:**
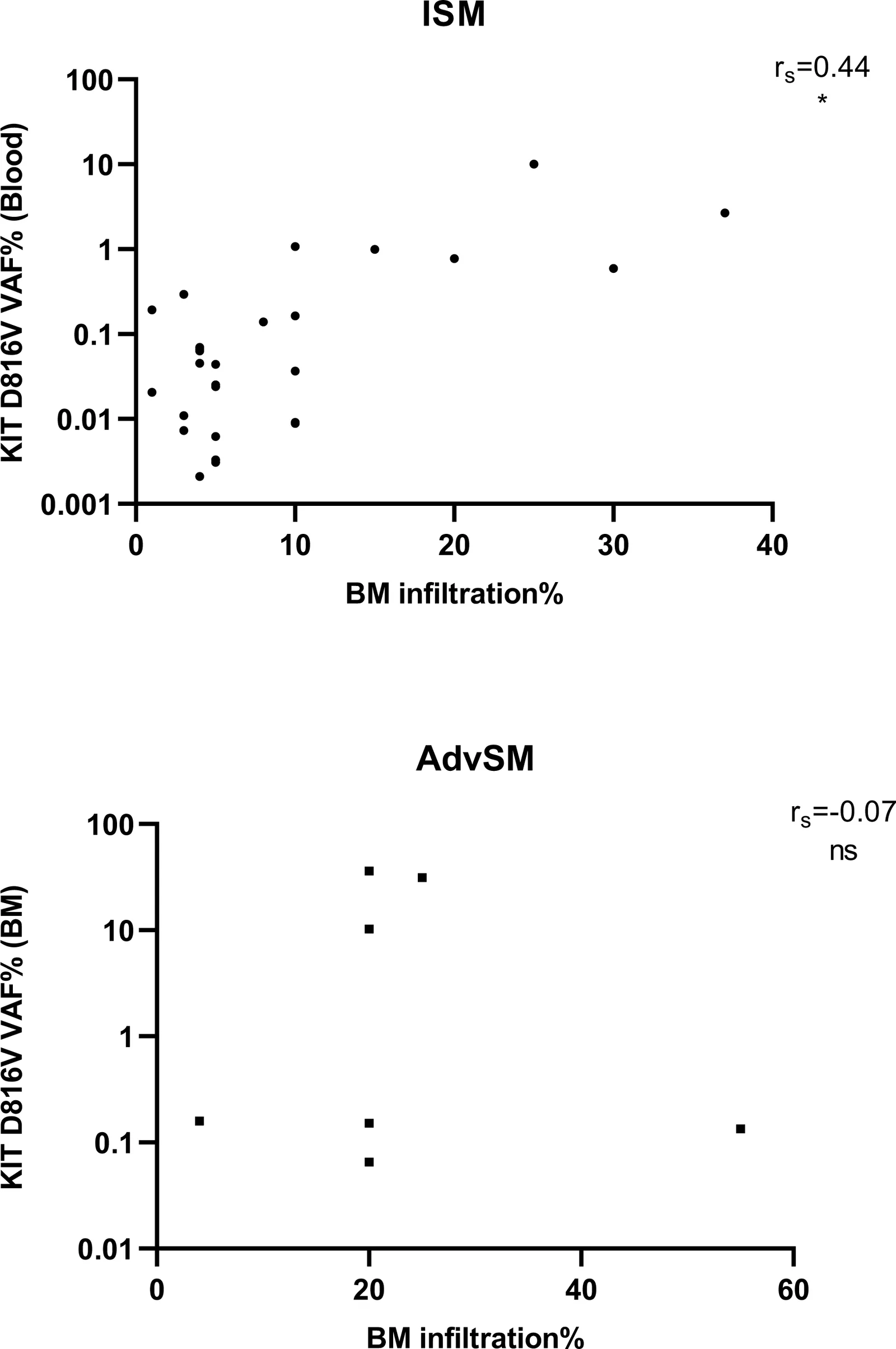


**Supplementary Figure S2:**